# Donor's therapeutic hypothermia vs. normothermia in kidney transplantation: a meta-analysis of randomized controlled trials

**DOI:** 10.3389/frtra.2025.1564460

**Published:** 2025-04-03

**Authors:** Luccas Marcolin Miranda, Pedro Emanuel Carneiro De Lima, Nathalia De Carvalho Dias Miranda, Giovanna Zaniolo Margraf, Juliano Riella

**Affiliations:** ^1^ Department of Medicine and Health Sciences, Pontifical Catholic University of Paraná, Curitiba, Paraná, Brazil; ^2^ Department of Medicine, Federal University of Latin American Integration, Foz do Iguaçu, Paraná, Brazil; ^3^ Department of Medicine, Bahiana’s School of Medicine and Public Health, Salvador, Bahia, Brazil; ^4^ Department of Surgery, Emory Transplant Center, School of Medicine, Emory University Atlanta, Atlanta, GA, United States

**Keywords:** delayed graft function, graft failure, graft survival, hypothermia, kidney transplantation

## Abstract

**Introduction:**

The shortage of organs remains one of the most challenging global problems nowadays. Donor's therapeutic hypothermia was suggested to decrease kidney delayed graft function (DGF) when compared to normothermia in previous trials, but the role of such intervention is still controversial. To assess this, we performed a systematic review and meta-analysis of randomized clinical trials (RCTs) investigating the benefits of donor hypothermia in DGF rate and Graft Failure.

**Methods:**

MEDLINE, Embase, and Cochrane databases were systematically searched for studies of deceased organ donors who underwent hypothermia or normothermia prior to kidney transplantation. Statistical analysis was performed using R Studio version 3.6. Heterogeneity was assessed using *I*^2^ statistics and a Baujat Plot.

**Results:**

Four different RCTs were analyzed, including more than 3,000 recipients. Donor hypothermia was associated with a lower, but not statistically significant, rate of DGF (RR 0.87; 95% CI 0.71–1.08; *P* = .21) and graft failure (RR 0.70; 95% CI 0.45–1.10; *P* = .12). When analyzing only expanded criteria donors, a significantly lower rate of DGF was observed in the hypothermia-treated group (RR 0.65; 95% CI 0.47–0.89; *P* = .008). Sensitivity analysis identified one study as an outlier, probably due to protocol deviation. When excluded from the analysis, a significant reduction in DGF rate was observed among the hypothermia-treated group (RR 0.80; 95% CI 0.67–0.94; *P* = .007).

**Conclusion:**

Our meta-analysis could not find a statistical difference between donor therapeutic hypothermia and normothermia in preventing DGF or Graft Failure. However, these results may be influenced by outliers and the limitations of the included studies. Further research is needed to clarify the role of donor hypothermia in kidney transplantation. If proven beneficial, it could be a promising alternative to sites where preservation techniques are limited, such as low-income countries.

**Systematic Review Registration:**

https://www.crd.york.ac.uk/PROSPERO/view/CRD42024581665, PROSPERO (CRD42024581665).

## 1 Introduction

The shortage of organ donors remains one of the most challenging global problems nowadays (1). Even though the raw number of transplants has doubled within the last three decades, the number of patients on the waiting list has increased six-fold (1). This demand has also been reflected in the donor profile. In fact, expanded criteria donors (ECDs) were introduced in an attempt to reduce graft shortages (2).

Within kidney transplantation, this change can be seen by the increase in the use of HIV-positive and HCV-positive donors, and by the rising number of donors with an elevated Kidney Donor Profile Index (KDPI) (1, 3). Additionally, delayed graft function (DGF), defined as the need for dialysis within the first week after transplant, has also followed this trend, increasing over the past decade (4). As a result, there has been growing scientific interest in transplant and organ preservation research (1).

In this context and guided by an apparent protective role of hypothermia on the renal function of patients with cardiac arrest, Niemann et al. conducted in 2015 a randomized clinical trial demonstrating the benefit of donor therapeutic hypothermia in preventing DGF (5, 6). Although additional trials were conducted, the role of donor hypothermia is still controversial within the current literature (2, 7–10).

Given this ongoing controversy, a systematic review and meta-analysis of the available randomized clinical trials was performed to assess whether therapeutic hypothermia could indeed decrease kidney DGF and graft failure rates.

## 2 Methods

This systematic review and meta-analysis was performed following the Cochrane Collaboration Handbook for Systematic Reviews of Interventions and the Preferred Reporting Items for Systematic Reviews and Meta-Analysis (PRISMA) guidelines.

### 2.1 Inclusion and exclusion criteria

Eligible studies included the following criteria: (1) Were Randomized Clinical Trials (RCTs); (2) Whose population was brain-dead deceased kidney donors; and (3) Compared donor's therapeutic hypothermia with normothermia. Furthermore, the studies were only included if they reported at least one of the outcomes of interest from this research: (1) Delayed graft function (DGF); (2) Graft failure or graft survival at 1 year; (3) Recipient mortality; or (4) Donor adverse events (hypotension, cardiac arrest, cardiac arrhythmias and/or systemic hypertension).

Additionally, studies were excluded from this research if: (1) Donors who underwent normothermia and therapeutic hypothermia were not randomized; or (2) The full paper was not available in English.

### 2.2 Search strategy

A systematic search was performed in PubMed (MEDLINE), EMBASE, and The Cochrane Central Register of Controlled Trials. The search block was built with a varied combination of the terms “kidney transplantation”, “hypothermia”, “deceased organ donor”, and their synonyms with Boolean operators. The Cochrane highly sensitive search strategies for the identification of randomized clinical trials were also included (6.4 Cochrane Handbook for Systematic Reviews of Interventions) (11). The protocol for this systematic review and meta-analysis was registered on PROSPERO (CRD42024581665).

### 2.3 Study selection and data extraction

Two authors (LMM and NDM) conducted an independent and blinded search. Abstracts were selected for full-text reading based on the inclusion criteria. The papers selected for full-text review were evaluated independently by each author, with results cross-checked. Any disagreements were addressed by the senior reviewer (JR). Moreover, the reference lists of all included studies were examined for any additional relevant titles.

Two researchers (LMM and PEL) independently extracted the data of interest from the included studies, which was then reviewed by the senior author (JR). The following data from individual studies were extracted: (1) study characteristics: study site, period, design, number of donors and recipients, follow-up time, population, and definition of hypothermia and normothermia; (2) donor characteristics: age, sex, height, weight, body mass index (BMI), proportion of standard and extended criteria donors (SCD and ECD respectively), prior treatment with hypothermia, creatine and estimated glomerular filtration rate at enrollment and before surgery, and kidney donor profile index (KDPI); (3) recipient characteristics: age, sex, height, weight, body mass index (BMI), donor-recipient weight ratio, positive hepatitis C virus (HCV), HLA mismatches, duration of RRT therapy before transplant, presence of previous renal transplant, and cold ischemia time; and (4) outcomes: delayed graft function (DGF), graft failure or survival, recipient mortality and donor adverse events.

The primary outcomes of this research were delayed graft function (DGF), defined as the need for dialysis within 1 week (7 days) of transplantation, and graft failure after 12 months, determined by allograft failure or dependency of renal replacement therapy (RRT). Donor's adverse events and recipients' mortality were addressed as secondary outcomes.

### 2.4 Bias assessment

The risk of bias assessment was conducted using Version 2 of the Cochrane risk-of-bias tool for randomized trials (RoB-2), following the recommendations from the Cochrane Handbook for Systematic Reviews of Interventions (11). Two authors (LMM and NDM) independently performed the evaluation, and disagreements were resolved through consensus. A study was categorized as having a high risk of bias if one or more domains assessed by the RoB-2 tool were rated as having a high or unclear risk.

### 2.5 Statistical analysis and subgroup analyses

Categorical results were expressed using Risk Ratios (RR) with 95% confidence intervals (95% CI). The Mantel-Haenszel test was used with a random effects model, and heterogeneity was assessed using Higgins and Thompson's I² statistic. An I² > 50% was considered significant for heterogeneity. Furthermore, the contribution of each trial to the overall heterogeneity was accessed through a Baujat Plot. Sensitivity analyses were performed for both primary outcomes by excluding each study from the outcome evaluation with the leave-one-out method. All statistical analyses were performed using R Studio version 3.6 software.

Finally, the subgroups addressed within this research were: (1) by country; (2) only ECD donors; and (3) by the adjunctive use of machine perfusion.

## 3 Results

**Figure 1:**
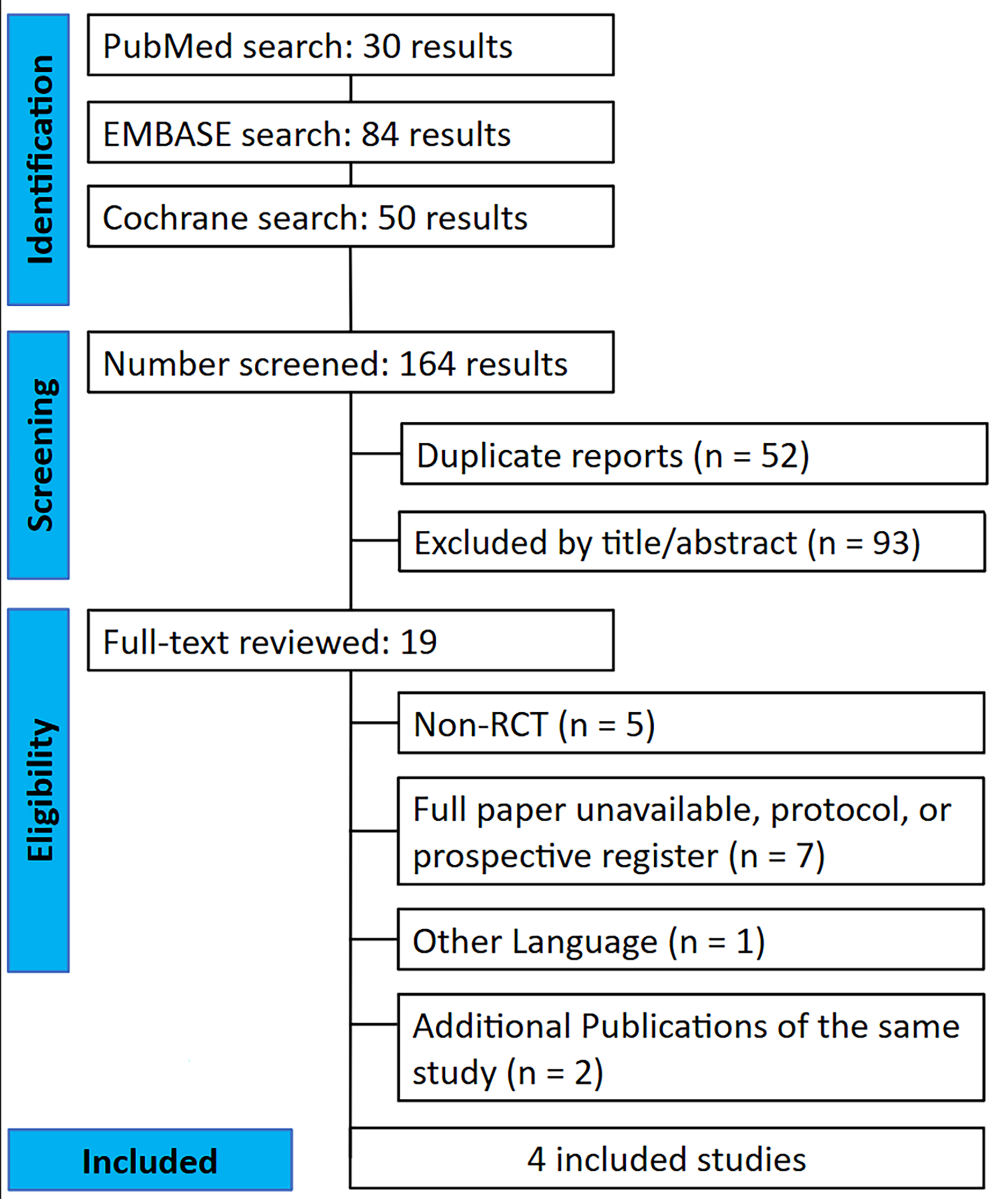
Study selection flow diagram.

The systematic search yielded 164 results. Following the removal of duplicates and the exclusion of studies that did not meet the eligibility criteria based on abstract screening, 19 studies were selected for full-text review. Of these, 4 studies, encompassing data presented in 6 articles, fulfilled the inclusion criteria and were incorporated into the analysis (Figure 1 and Table 1).

**Table 1:** Baseline characteristics of included studies.

Characteristic	Malinoski et al. (10)	NMA 2015–2019^a^	Patel et al. (9)	HYPOREME (2)
Included studies’ characteristics				
Design	RCT, Multicenter	RCT, Multicenter	RCT, Multicenter	RCT, Multicenter
Country	USA	USA	USA	France
ECD definition	Expanded criteria were a declaration of brain death according to hospital criteria for neurologic determination of death and an age of more than 59 years or an age of 51–59 years with at least two of the following coexisting illnesses: chronic hypertension, death resulting from a cerebral vascular accident, or a serum creatinine level of more than 1.5 mg per deciliter.	Expanded criteria are based on established definitions that include the donor's age, the presence or absence of hypertension, the baseline creatinine level, the cause of death, and whether or not the donor had received hypothermia therapy before declaration of death according to neurologic criteria	Specifically, in the assessment of a deceased donor kidney quality, traditional risk factors included history of hypertension, donor age older than 60 years, terminal serum creatinine level greater than 1.5 mg/dl and/or death from a cerebrovascular accident based on which a kidney was deemed lower risk (SCD) or higher risk (ECD)	Eligible expanded criteria donors were older than 60 years or were aged 50–59 years and had at least two other risk factors (history of hypertension, creatinine >1.5 mg/dl, or cerebrovascular cause of death)^d^
Donor's baseline characteristics				
Number of donors Hypo/Normo	479/510	150/152	236/245	142/156
Adjunctive HMP (%) Hypo/Normo	100/100	0/0	0/0	89/90
Age—years Hypo/Normo	42 ± 14/42 ± 13	45 ± 15/45 ± 15	34.52 ± 11.62/33.92 ± 10.6	70.9 ± 9.4/71.8 ± 8.7
Female Hypo/Normo	180 (38%)/191 (37%)	56 (37.3%)/56 (36.8%)	86 (36%)/90 (37%)	72 (51%)/75 (48%)
BMI	29 ± 7/30 ± 8	28.9 ± 6.8/29.3 ± 7.3	28.24 ± 6.36/27.15 ± 6.61	25.5 ± 4.6/26.6 ± 4.8
ECD Hypo/Normo	98 (20%)/107 (21%)	40 (26.7%)/41 (27.0%)	4 (2%)/5 (2%)	142 (100%)/156 (100%)
KDPI Hypo/Normo	46.36 ± 29.22/47.95 ± 28.74	51 ± 29/53 ± 28^b^	28.32 ± 21.9/28.99 ± 20.46	91.0 ± 11.2/91.4 ± 9.8
Last Creatinine before Transplant/Procurement—mg/dl Hypo/Normo	1.19 ± 1.03/1.44 ± 1.35	1.1 ± 0.8/1.2 ± 0.8	0.86 ± 0.49/1.06 ± 0.87	0.95 ± 0.53/0.98 ± 0.53^d^
Recipient's baseline characteristics				
Number of recipients Hypo/Normo	479/510	280/286	460/474	251/275
Age—years Hypo/Normo	51 ± 15/52 ± 14	52.3 ± 13.5/53.4 ± 15.4^b^	48.13 ± 15.03/47.32 ± 15.62	65.8 ± 10.4/65.9 ± 10.2
Female Hypo/Normo	194 (41%)/192 (38%)	91 (38.2%)/101 (42.4%)^b^	191 (42%)/201 (42%)	106 (42%)/97 (35%)
BMI Hypo/Normo	28 ± 9/28 ± 6	27.2 ± 5.3/26.9 ± 5^b^	28.27 ± 10.69/27.79 ± 7.61	26.4 ± 4.4/26.2 ± 4.1
Donor to recipient weight ratio Hypo/Normo	1.2 ± 0.5/1.2 ± 0.5	1.20 ± 0.50/1.25 ± 0.71^b^	1.16 ± 0.61/1.14 ± 0.73	NA
Positive HCV Hypo/Normo	14 (3%)/25 (5%)	10 (4.3%)/15 (6.5%)^b^	18 (4%)/18 (4%)	4 (2%)/0 (0%)
HLA mismatches Hypo/Normo	4.1 ± 1.5/4.2 ± 1.4	4 ± 2/4 ± 2^b^	4.2 ± 1.43/4.0 ± 1.62	4.3 ± 1.5/4.2 ± 1.8
Duration of RRT before transplantation—days Hypo/Normo	1,680 ± 1,146/1,692 ± 1,353	2,061 ± 1,375/2,030 ± 1,523^c^	1,491.85 ± 1,223.42/1,487.43 ± 1,213.72	1,241 ± 1,058.5/1,350.5 ± 1,241^c^
Previous renal transplant Hypo/Normo	60 (13%)/55 (11%)	26 (11.1%)/26 (10.9%)^b^	54 (12%)/60 (13%)	37 (15%)/56 (20%)
Cold–ischemia time–h Hypo/Normo	19.1 ± 8.0/19.3 ± 8.3	13.9 ± 7.3/15.6 ± 8.3^b^	15.45 ± 7.63/15.99 ± 7.9	15.5 ± 5.6/16.3 ± 5.8

Data are reported as mean ± SD or No. (%). BMI, body-mass index is the weight in kilograms divided by the square of the height in meters; ECD, expanded criteria donors; GFR, glomerular filtration rate; HCV, hepatitis C virus, HLA, human leukocyte antigen; KDPI, kidney donor profile index is a cumulative percentage scale that represents an overall estimate of the risk of graft failure for an individual kidney. Scores range from 0% to 100%, with higher values indicating greater risk. RRT, renal replacement therapy. For additional information concerning baseline characteristics of included studies, donors and recipients refer to Supplementary Table S1.

^a^ NMA, Niemann (5), Malinoski (20), Axelrod (22), which were three different publications of the same research.

^b^ Data not available for all patients.

^c^ Originally reported as years and converted to days by multiplying the original value by 365.

^d^ Originally reported in µmol/L, converted to mg/dl by dividing the original value by 88.42 and rounding to two decimal cases.

Donors in all studies underwent therapeutic hypothermia (34°C–35°C) in the intervention group, while donors in the control group were maintained at normothermia (36.5°C–37.5°C). Of the four studies included in this research, three were conducted in the USA and one in France. The study population contained both SCD and ECD for all studies except for the HYPOREME trial whose population embraced only ECD donors (2). The percentage of females varied between 36% and 51% among kidney donors and 35%–42% among recipients. Mean age varied between 33.92 and 71.8 years between donors and 47.32–65.9 years among recipients. Only two papers included donors whose kidneys underwent adjunctive use of machine perfusion (2, 10). A summary of the studies and the characteristics of donors and recipients is available in Table 1 (Refer to Supplementary Tables S1, S2 for additional information).

Only one study was classified as having a high risk of bias (Supplementary Table S3) due to identified biases within the protocol deviation domain (10). Given the high level of heterogeneity observed, a random-effects model was applied to all statistical analyses.

### 3.1 Delayed graft function

**Figure 2:**
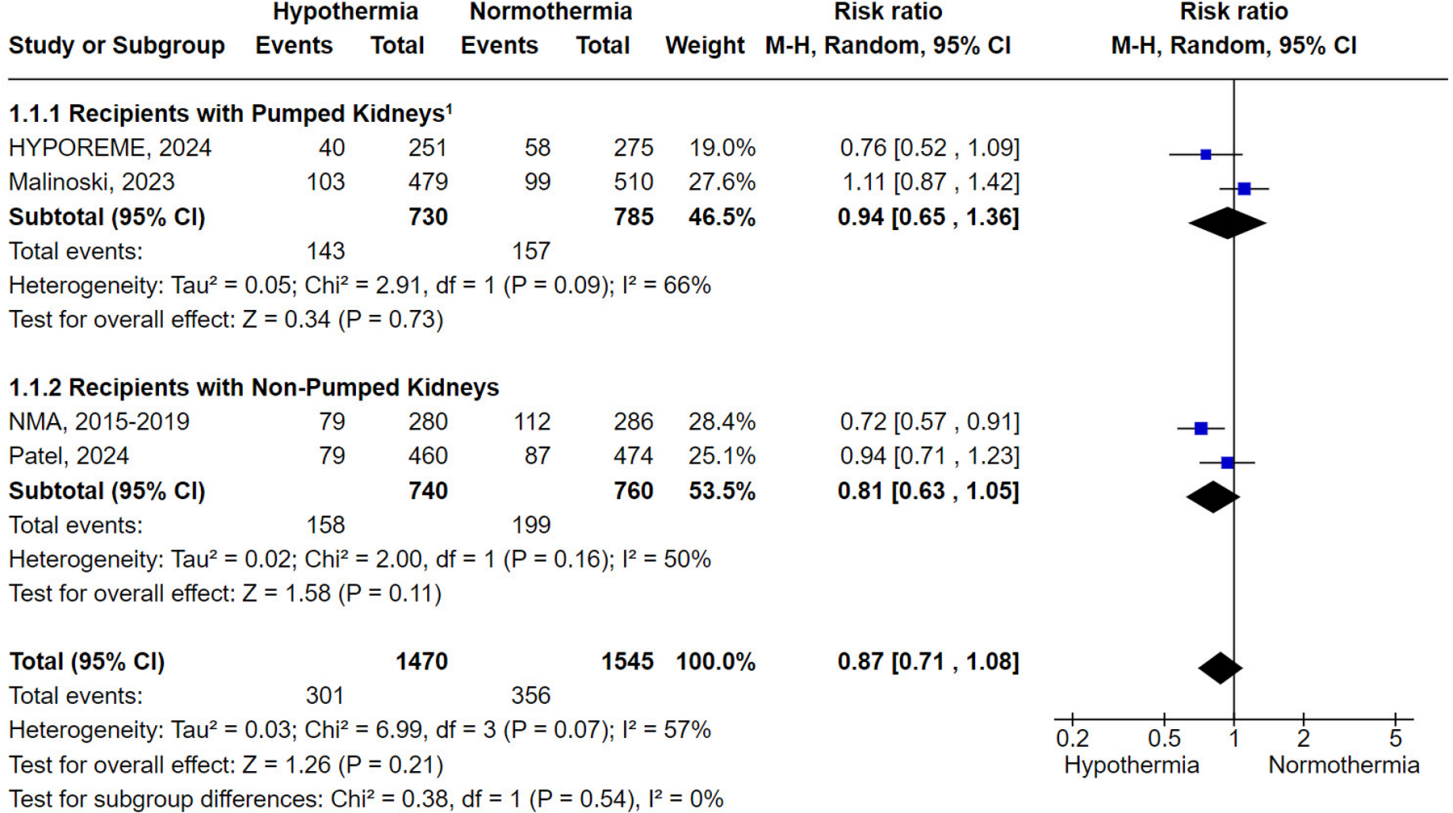
Delayed graft function with donor's therapeutic hypothermia vs. normothermia stratified by subgroup analysis involving the adjunctive use of machine perfusion. ^1^Around 10% of the kidneys in the HYPOREME trial were not submitted to machine perfusion, being a significant proportion due to severe atherosclerotic disease or unfavorable anatomy. Since these factors are already associated with poorer DGF outcomes and the subdivision of the trial's population would distort sample size calculation, the HYPOREME trial was analyzed in an intention-to-treat manner concerning the use of machine perfusion.

DGF was reported in all four trials included. Donor hypothermia led to a numerically lower, but not statistically significant, rate of DGF (RR 0.87; 95% CI 0.71–1.08; *P* = .21) (Figure 2).

**Figure 3:**
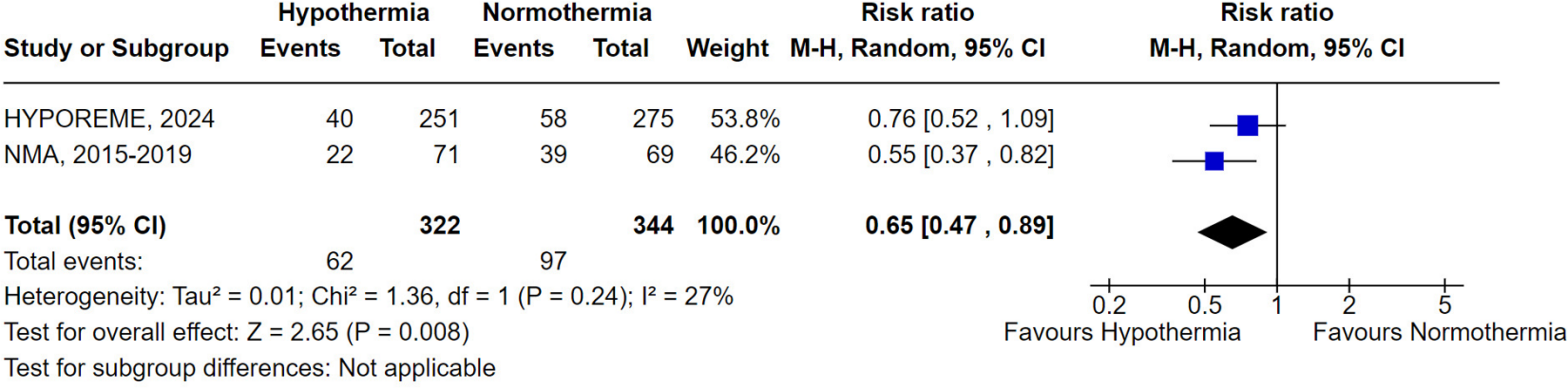
Delayed graft function with donor's therapeutic hypothermia vs. normothermia including only extended criteria donors.

Subgroup analyses based on the adjunctive use of machine perfusion reached similar results for non-pumped and pumped kidneys (RR 0.81; 95% CI 0.63–1.05; *P* = .11 and RR 0.94; 95% CI 0.65–1.36; *P* = .73, respectively) (Figure 2). Similarly, the subgroup analysis limited to studies conducted in the USA did not achieve statistical significance (RR 0.90; 95% CI 0.70–1.17; *P* = .45) (Supplementary Figure S1). In contrast, the subgroup analysis focusing exclusively on ECDs demonstrated that hypothermia was statistically superior to normothermia in preventing DGF (RR 0.65; 95% CI 0.47–0.89; *P* = .008) (Figure 3).

### 3.2 Graft failure or renal replacement therapy (RRT)

**Figure 4:**
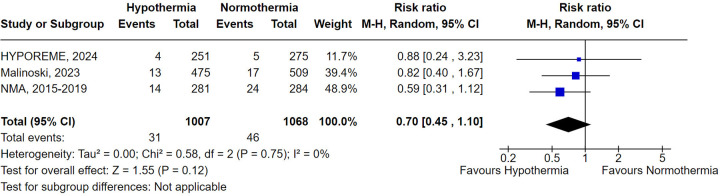
Graft-Failure with donor's therapeutic hypothermia vs. normothermia.

Graft failure or necessity of RRT in 12 months was reported in 3 trials. Similar to DGF, therapeutic hypothermia was associated with a lower, but not statistically significant, incidence of graft failure or RRT dependency after 12 months (RR 0.70; 95% CI 0.45–1.10; *P* = .12) (Figure 4).

Due to limited data, only the subgroup analysis involving studies conducted in the USA was performed. Consistent with the overall analysis, hypothermia led to a non-statistically significant reduction in graft failure and RRT necessity (RR 0.68; 95% CI 0.42–1.10; *P* = .12) (Supplementary Figure S2).

### 3.3 Secondary outcomes

Mortality outcomes were reported in two trials, while adverse events were documented in three of the four included studies (Supplementary Figures S3, S4). Neither the analyses of mortality nor adverse events reached statistical significance. Hypothermia was associated with a non-statistically significant decrease in recipient mortality (RR 0.91; 95% CI 0.58–1.43; *P* = .69) and an increase in donor adverse events (RR 1.28; 95% CI 0.45–3.62; *P* = .64).

### 3.4 Sensitivity analysis

**Figure 5:**
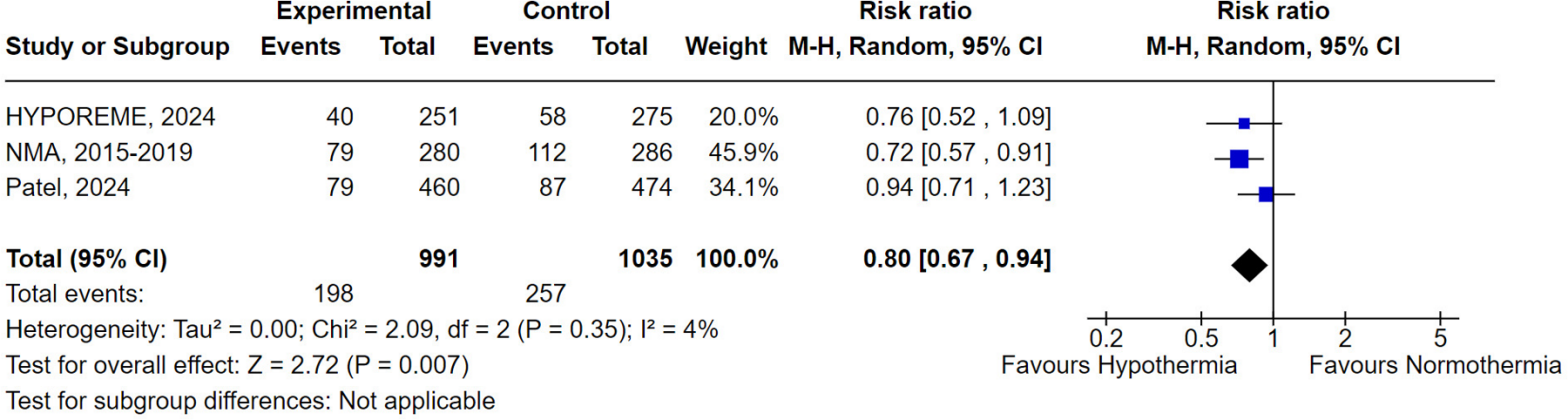
Delayed graft function with donor's therapeutic hypothermia vs. normothermia excluding Malinoski et al. (2023) (10).

When Malinoski et al. (2023) study (10), classified with a high risk of bias in the RoB-2 tool, was excluded from the analysis, donor hypothermia was associated with a statistically significant lower rate of DGF when compared to normothermia (RR 0.80; 95% CI 0.67–0.94; *P* = .007) (Figure 5). Additionally, the exclusion of this study also led to a non-significant heterogeneity (*I*^2^ = 4%). This observation is consistent with the Baujat Plot analysis which demonstrated that the study not only contributed disproportionately to the overall heterogeneity, occupying the highest value in the *x*-axis, but was also the study that most contributed to the overall pooled result (Supplementary Figure S5).

The remaining sensitivity analyses for DGF and graft failure reached similar results to the overall pooled analyses, demonstrating a numerically, but not statistically significant, benefit of hypothermia in decreasing the rate of both DGF and graft failure (Supplementary Figures S6, S7).

## 4 Discussion

The use of hypothermia as a therapeutic approach is not new. The induction of hypothermia dates back to the 1940s, when patients were routinely treated for cardiac arrest and traumatic brain injury with deep hypothermia (<30°C) (12). As expected, significant problems arose and the interest in hypothermia fizzled out until the 1980s (12). The positive results observed at that time, coupled with the understanding that mild hypothermia (31°C–35°C) could improve neurological outcomes without having many side effects, rekindled the interest of researchers in the topic (12).

Today, known benefits of hypothermia include the interruption of the apoptotic pathway, suppression of ischemic and ischemic-reperfusion immune responses, and reduction of free-radical production, events that are often present in DGF (12, 13). In fact, the pathophysiology of DGF, which resembles that of acute tubular necrosis, occurs due to a variety of processes that could be mitigated by hypothermia, including immunological reactions, endothelial damage, and ischemic injury (12, 13). However, despite these known benefits, the effect of donor hypothermia on renal function is still unclear (5).

Previous cohorts nested in randomized clinical trials have observed an association between spontaneous donor hypothermia and lower creatinine levels prior to organ procurement (7, 14). Furthermore, a study involving patients with cardiac arrest has suggested a potential protective effect of mild hypothermia on renal function (6). Still, randomized clinical trials were not conclusive in addressing this benefit, and the role of donor hypothermia in kidney transplantation remains uncertain.

To our knowledge, this is the first meta-analysis to address this topic. The analysis, including over 3,000 patients, revealed a numerical reduction in DGF and graft failure rates, though these findings were not statistically significant. However, after excluding Malinoski et al. (10), identified as an outlier in the sensitivity analysis, the decrease in DGF rates associated with donor hypothermia reached statistical significance and heterogeneity was significantly reduced.

A possible explanation for such a phenomenon would be the high risk of bias of the included outlier. Malinoski's work originally aimed to compare isolated hypothermia with hypothermic machine perfusion (HMP) or combination therapy. However, 27% (269/989) of the intended patients did not use hypothermic machine perfusion. To avoid selection bias, an intention-to-treat analysis was conducted (15). However, the study does not provide information on the distribution of these 269 patients across the study groups, which justifies its classification under the high-risk of bias category in the protocol deviation domain of the RoB-2 tool (16). Since our meta-analysis intended to compare normothermia and hypothermia in donors, the analyses included the study groups utilizing combination therapy and isolated machine perfusion. Therefore, unequal distribution of patients who did not undergo machine perfusion could introduce bias into our analysis, given the well-established benefit of HMP in preventing DGF (16). This imbalance may potentially explain why Malinoski's study was the only one to favor normothermia over hypothermia in preventing DGF. Furthermore, supporting our hypothesis, is a recent Cochrane review examining the role of normothermic and hypothermic machine perfusion in kidney transplantation (16). The review has also highlighted the potential bias of Malinoski's work, classifying it under the high-risk of bias category as well (16).

The use of machine perfusion (MP) dates back to 1968 when Belzer et al. successfully preserved a human kidney using HMP (17). However, the machine's considerable size posed significant challenges for transportation (16). Nowadays, MP technologies have evolved and differ substantially, including continuous modalities where perfusion is maintained during transport, or end-ischemic when the therapy is initiated at the implanting center after a period of cold storage during transport (16). Furthermore, MP techniques also vary in terms of oxygen provision (oxygenated vs. non-oxygenated) and flow patterns (pulsatile vs. non-pulsatile) (16).

Currently, guidelines recommend the use of HMP which is considered to be the standard of care for deceased kidney donors (18). Known benefits of HMP include lower rates and reduced duration of DGF, as well as improved overall graft survival when compared to static cold storage (16, 19). Moreover, these benefits are supported by robust up-to-date evidence, originating from well conducted RCTs and a large meta-analysis (16).

To address the possible influence of machine perfusion techniques in the context of hypothermia, we conducted subgroup analyses evaluating DGF in both pumped and non-pumped kidneys (Figure 5). Despite a clear graph skew demonstrating lower DGF rates in the hypothermia-treated group when considering only kidneys that have not undergone HMP, there was no statistical difference between the intervention and control. The same was true considering the analysis of the pumped kidneys, although hypothermia appeared to have a lesser impact on this population compared to the non-pumped organs. However, caution should be taken when interpreting such results given the possible lack of sufficient data, as only two trials were included within each analysis.

It is well established that extended criteria donors (ECDs) are often associated with inferior outcomes as the chronic conditions commonly present in these donors increase the risk of DGF (5). This elevated risk is driven by various physiological factors including a pro-inflammatory environment, oxidative stress, and ischemic injury which can be mitigated by the application of hypothermia (5, 12, 13, 20). Consequently, ECDs may, in theory, derive greater benefit from this therapy (5, 12, 13, 20). Our subgroup analysis supports this hypothesis; however, caution is warranted, as only two trials reported DGF events within this specific subgroup.

Due to the global organ shortage, great effort is being made to develop strategies to optimize organ usage without negatively affecting outcomes. The DONORS trial, protocolized in 2019, is a Brazilian multi-site cluster randomized controlled trial that proposes to assess the benefit of an evidence-based bedside checklist with goals and recommendations for the management of brain-dead organ donors (21). The trial suggests that clinical management strategies focused on hemodynamic stabilization, optimal ventilatory support, and temperature control may enhance organ quality and lead to improved clinical outcomes for transplant recipients (21).

In this context, if proven beneficial, donor therapeutic hypothermia could be a promising strategy as it is an easily feasible, low-cost intervention (2, 5, 7, 20, 22). Furthermore, even if its benefits are less pronounced or outweighed by those of machine perfusion, incorporating donor hypothermia into temperature control management strategies could be valuable as perfusion devices are not widely available in all transplant centers. In fact, machine perfusion is used in only 32%–38% of all kidneys considered for transplantation in the United States, and is rarely available in developing countries (10). Moreover, donor hypothermia may also lead to additional benefits beyond the potential reduction in DGF rate. Hypothermia was associated with statistically significant lower serum creatinine concentration and a higher estimated glomerular filtration rate (eGFR) at all timepoints evaluated in the HYPOREME trial, for example (2). Although not meta-analyzed, as this was reported in a single trial, these results are of clinical significance as several studies have already linked impaired kidney graft function at 1 year with increased risk of graft failure and cardiovascular death (23–26).

It is important to note, however, that Malinoski's trial, despite being limited by biases, has demonstrated that undergoing HMP when appropriate leads to lower rates of DGF when compared to undergoing hypothermia alone (10). Therefore, the value of therapeutic hypothermia in kidney transplantation is probably as an adjunctive therapy to machine perfusion, which should only be considered as an alternative to perfusion devices when these are not available. This approach is of particular interest in low-income countries, where access to perfusion devices may be limited.

Another important factor to consider is that donor therapeutic hypothermia is not without potential adverse effects. It can increase donor-related complications including cardiac arrest and other cardiovascular events, which are associated with organ loss (7, 21). Our findings did show a numerical increase in these events, but the difference was not statistically significant. Likewise, none of the trials that have evaluated the number of individual or total organs transplanted from each donor have reported significant differences between study arms, being the overall failure of non-kidney organ transplants also similar across the study groups (5, 10, 20). In addition, donor hypothermia does not seem to be associated with decreased urine output, hemodynamic instability, or elevated lactate blood concentration which could potentially negatively affect organ utilization and graft outcomes (2, 10).

Regarding rejection rates, these were reported in a single trial and, as a result, could not be meta-analyzed as initially planned (2). Nevertheless, the results from that trial showed no statistical differences between the intervention and control (2). Similarly, our meta-analysis has also not found a significant difference in recipient mortality between donor hypothermia and normothermia (Supplementary Figure S3), suggesting that donor therapeutic hypothermia is probably a safe intervention and that its benefits may outweigh its risks.

Despite the substantial interest in organ preservation research, very few studies explore interventions in donors (1, 5). These may reflect logistical and ethical aspects that surround donor-intervention research and the subsequent use of such organs (27, 28). A waitlist candidate offered a compatible organ that has been exposed to an intervention, for instance, might decline it specifically as a result of that exposition, while candidates who accept such organs will be directly exposed to the associated risks, thus making it difficult to carry out research exploring donor's interventions (27, 28). Without large high-quality trials, in turn, it is difficult to address the clinical benefits and risks of these potential interventions.

Moreover, although legally, research conducted on deceased donors does not require consent or ethics review committee approval, the intervention is rarely without any risk, raising a clear ethical issue that surrounds the implementation of interventions, such as hypothermia, in this type of donor (27, 28). Therefore, although donor therapeutic hypothermia is an easily feasible low-cost intervention in which benefits may outweigh the risks, logistical and ethical concerns may pose important barriers to its widespread clinical adoption.

### 4.1 Limitations

Our meta-analysis has several limitations. Firstly, when analyzing the references of the full-text screened articles, the work of Kepu et al. was identified (29). This work randomly assigned 38 donors to therapeutic hypothermia or normothermia and included DGF as an observed outcome (29). The results section in the abstract mentioned a significantly lower DGF rate within the hypothermia-treated group (6%) when compared to normothermia (24%) (29). However, the article was not included in the present meta-analysis, as only the abstract was available in English. Therefore, we recommend expanding further research in order to assess non-English studies.

Moreover, although included studies defined hypothermia and normothermia groups equally, cooling protocols differed among the studies, and even within the same trial. Different cooling methods vary in terms of efficiency and safety, with intravascular cooling, gel pads, and water-circulating blankets being more effective compared to conventional cooling and air-circulating blankets, for example (30). This variability could introduce a bias that may influence the results of this analysis.

Thirdly, the role of hypothermia in the context of new technologies including normothermic machine perfusion (NMP) and normothermic regional perfusion (NRP) should also be investigated. In fact, portable NMP technologies have now entered phase 1 trials (16). Despite the non-significant benefit of NMP when compared to static cold storage observed in a recent RCT (31), a recent systematic review outlined the feasibility and possible benefit of both NMP and NRP within donors after circulatory death (DCD), which were also not included in our analysis (32). Moreover, hypothermic machine perfusion techniques also vary significantly, potentially impacting graft outcomes (16). Therefore, to effectively evaluate outcomes such as graft failure, ensuring equivalence between the machine perfusion techniques used is recommended.

Furthermore, although several sub-analyses were performed to address subgroups of interest, it was impossible to perform an analysis of DGF including only SCDs. Only one trial has reported DGF separately for standard criteria donors and the study has terminated early, making it difficult to draw conclusions on the impact of hypothermia among this population (5). Similarly, other subpopulations of interests such as HIV-positive donors and recipients transplanted after the HIV Organ Policy Equity (HOPE) Act and HCV-positive patients were also not addressed due to the lack of sufficient data.

Finally, only four randomized trials were included in this analysis, highlighting the need for further studies to more accurately address the role of donor's hypothermia in kidney transplantation.

### 4.2 Recommendations for future research

An important limitation of the present meta-analysis is the lack of standardization in the design and reporting of included trials. Because of this, the authors have prepared a list of recommendations in an attempt to reduce the biases and assure comparability of future trials. These recommendations are summarized in Table 2.

**Table 2:** Recommendations for future trials.

Cooling methods
Cooling methods should be described and comparable across study groups
The cooling method used should be decided on a basis of efficiency, safety and availability
Intravascular cooling appears to be the most efficient method in inducing and maintaining therapeutic hypothermia, so its use is recommended
Water-circulating blankets and gel-pads are other valid efficient alternatives to intravascular cooling
Conventional cooling and air-circulating blankets seem to be inferior to intravascular cooling, gel-pads and water-circulating blankets and therefore should be avoided when other methods are available
If there is great variability in the cooling methods utilized, consider performing a sub-analysis stratifying outcome results per cooling method used
Core temperature measurement
Core temperature measurement should be described and comparable across study groups
Core temperature measurement methods should be decided on a basis of accuracy, clinical context and time lag between site and gold-standard, requiring the clinicians to also account for possible complications, overcooling or technical failure of the chosen method
The gold standard for core temperature measurement uses a pulmonary artery catheter
Valid alternative sites also recommended for core temperature measurement include the bladder, esophagus and the rectum
Use of machine perfusion
Use of Machine Perfusion should be comparable across study groups
The type of perfusion device used should be clearly stated. Flow pattern used (pulsatile vs. non-pulsatile) and the oxygen provision (oxygenated vs. non-oxygenated) of the perfusion device should be described
Authors should also report whether the perfusion was maintained during transport or initiated at the transplanting center after a period of cold storage during transport
Reporting and stratification of results
The analysis of the following outcomes of interest is recommended: (1) DGF; (2) Graft Failure; (3) Rejection Rates; (4) Serum Creatinine Concentration and eGFR; (5) Adverse Events; (6) Recipient Mortality; and (7) Number of individual and/or total organs transplanted per donor
Outcome results should preferably be stratified according to donor type (ECD vs. SCD) and machine perfusion usage (pumped vs. non-pumped)

Firstly, it is important to assure comparability of the cooling methods used across study groups as this may have a direct impact in graft outcomes. Cooling techniques that can effectively lead to rapid hypothermia induction, for example, can reduce the risks of short-term side effects such as shivering and metabolic disorders (33). In this context, intravascular cooling seems to be the most efficient cooling method for both inducing and maintaining therapeutic hypothermia (30, 33, 34). These benefits, however, must be weighed against the potential risks and barriers of this cooling method, including the necessity of an invasive procedure and its procedural risks (33). Other reliable alternatives are gel pads and water-circulating blankets, non-invasive techniques that show an overall similar efficiency to intravascular cooling (30, 33, 34). Conversely, conventional cooling and air-circulating blankets seem to be inferior to intravascular cooling, gel pads, or water-circulating blankets, and should be avoided when more reliable methods are available (30, 33, 34). The choice of a particular cooling method should be made by the clinician considering efficiency, safety and availability. If great variability is present, we advise conducting a sub-analysis stratifying outcome results per cooling method used.

Furthermore, the core temperature measurement method utilized should also be similar across study groups. The gold-standard site for measuring the core temperature is the pulmonary artery, but this method requires an invasive and complex insertion procedure (33, 35, 36). Alternatively, measuring the core temperature in the bladder, esophagus or rectum is less invasive and still provides reliable temperature measurements (33, 35, 36). In choosing the measurement site, it is important to consider factors such as accuracy, team expertise, and the limitations inherent to each technique.

Thirdly, considering the benefits of perfusion devices, we suggest stratifying outcome results by the use of machine perfusion. Additionally, we also recommend describing the type of perfusion devices used in respect to flow pattern (pulsatile vs. non-pulsatile) and oxygenation (oxygenated vs. non-oxygenated). Currently, there is limited data and no consensus regarding the optimal flow pattern or if oxygenated machine perfusion is superior to non-oxygenated devices, but this may be of importance when interpreting results in a near future (37–40). Moreover, it is also important that studies describe if the perfusion was maintained during transport or initiated at the transplanting center after a period of cold storage. This is often not reported, and not only might be of clinical interest, but give important insights on studies heterogeneity (16).

Lastly, the inclusion of additional outcomes, aside from donor adverse events, graft failure, recipient mortality, and DGF is also strongly recommended. Analyzing other potential benefits of hypothermia such as higher eGFR, and additional risks such as rejection rates, would provide important clinical evidence to better understand the advantages and limitations of donor hypothermia in kidney transplantation. Similarly, stratifying outcome results per donor type (ECD vs. SCD) is also recommended given the potential different impacts of therapeutic hypothermia within these two populations.

## 5 Conclusion

Our meta-analysis did not find a statistical difference between normothermia and therapeutic hypothermia in preventing DGF or graft failure. However, these results may be influenced by outliers and the limitations of the included studies. Further research is needed to better understand the role of therapeutic hypothermia in DGF rates, preferentially controlling for the cooling methods used, and for the adjunctive usage of machine perfusion techniques. Further analysis including non-English studies is also recommended. If proven beneficial, it could serve as a promising alternative to sites with limited access to other preservation techniques.

## Data Availability

The original contributions presented in the study are included in the article/Supplementary Material, further inquiries can be directed to the corresponding author.
