# Supplementary material for: Donor's therapeutic hypothermia vs. normothermia in kidney transplantation: a meta-analysis of randomized controlled trials
Source: Front Transplant. 2025 Apr 3;4:1564460. doi: 10.3389/frtra.2025.1564460 (PMC12003384; doi:10.3389/frtra.2025.1564460)
Supplement: Supplementary file 1 [file Datasheet1.pdf]

SUPPLEMENTARY MATERIAL

Title: Donor's Therapeutic Hypothermia vs Normothermia in Kidney Transplantation: A Meta-analysis of Randomized Controlled Trials

Authors: Luccas Marcolin Miranda[1], Pedro Emanuel Carneiro De Lima[2], Nathalia De Carvalho Dias Miranda[3], Giovanna Zaniolo Margraf[1], Juliano Riella[4]

Affiliations:

[1]Pontifical Catholic University of Paraná, Department of Medicine and Health Sciences;

[2]Federal University of Latin American Integration, Department of Medicine;

[3]Bahiana's School of Medicine and Public Health, Department of Medicine;

[4]Emory Transplant Center, Department of Surgery, School of Medicine, Emory University Atlanta, Georgia, USA

Correspondence to:

Luccas M. Miranda: lucc.marcolinmiranda@gmail.com – Street Imaculada Conceição, 1155 – Prado Velho, Curitiba, Paraná, Brazil – ORCID ID: https://orcid.org/0009-0005-8885-0409

Table of Contents

Methods Supplement

Search Strategies:

Adverse Events per Study Group:

Table S1 – Donor’s Adverse Events per Study

Results Supplement

Additional Information of Included Studies, Donors and Recipients:

Table S2 - Additional Information not reported in Table 1

Primary Outcomes Additional Subgroup Analyses:

Figure S1 – Delayed graft function (DGF) with Donor’s Therapeutic Hypothermia vs Normothermia including only USA studies

Figure S2 – Graft Failure with Donor’s Therapeutic Hypothermia vs Normothermia including only USA studies

Secondary Outcomes Analyses:

Figure S3 – Recipient’s Mortality with Donor’s Therapeutic Hypothermia vs Normothermia

Figure S4 – Donor’s Adverse Events with Donor’s Therapeutic Hypothermia vs Normothermia

Heterogeneity Analysis

Figure S5 – Contribution to Overall Heterogeneity – DGF Baujat Plot

Sensitivity Analyses – Leave-one-out method:

Figure S6 – Sensitivity Analysis (Leave-one-out) - DGF

Figure S7 – Sensitivity Analysis (Leave-one-out) – Graft Failure

Risk of Bias Assessment:

Table S3 – RoB-2 Bias Assessment of Included Studies

Methods Supplement

Search Strategies:

In accordance with the recommendations from the 6.4 Cochrane Handbook for Systematic Reviews of Interventions we decided to use the Cochrane Highly Sensitive Search Strategy to validly identify, with sufficient sensitivity, randomized clinical trials available in MEDLINE and EMBASE databases.

PubMed (MEDLINE): ("Kidney Transplantation" OR "Kidney graft" OR "Renal graft" OR "Renal Transplantation" OR "Kidney Transplant Surgery" OR "Renal Transplant" OR "Kidney Transplant" OR "Kidney Transplantation"[Mesh] OR "Kidney Donor*") AND ("termed targeted temperature management" OR "targeted temperature management" OR "Therapeutic Hypothermia" OR Hypothermia OR "Hypothermia, Induced"[Mesh] OR "Cooling therapy") AND (DBD OR DCD OR "Deceased Donor*" OR" OR "Deceased Organ Donor*" OR "Deceased Kidney donor* OR "Deceased renal donor*" OR "Brain-dead donor*" OR "Brain-dead Kidney Donor*" OR "Brain-dead Organ Donor*" OR "Brain-dead renal donor*" OR "Non-living donor*" OR "Non-living kidney donor*" OR "Non-living renal donor*" OR "Non-living organ donor*" OR "Cadaveric donor*" OR "Cadaveric kidney donor*" OR "Cadaveric Renal donor*" OR "Cadaveric Organ donor*" OR "Tissue Donors"[Mesh]) AND ("randomized controlled trial" [pt] OR "controlled clinical trial" [pt] OR randomized [tiab] OR placebo [tiab] OR "drug therapy" [sh] OR randomly [tiab] OR trial [tiab] OR groups [tiab])

EMBASE: ('kidney transplantation' OR 'kidney graft' OR 'renal graft' OR 'renal transplantation' OR 'kidney transplant surgery' OR 'renal transplant' OR 'kidney

transplant' OR 'kidney transplantation'/exp OR 'kidney donor*') AND ('termed targeted temperature management' OR 'targeted temperature management' OR 'therapeutic hypothermia' OR 'hypothermia' OR 'induced hypothermia'/exp OR 'cooling therapy') AND ('dbd' OR 'dcd' OR 'deceased donor*' OR 'deceased organ donor*' OR 'deceased kidney donor*' OR 'deceased renal donor*' OR 'brain-dead donor*' OR 'brain-dead kidney donor*' OR 'brain-dead organ donor*' OR 'brain-dead renal donor*' OR 'non-living donor*' OR 'non-living kidney donor*' OR 'non-living renal donor*' OR 'non-living organ donor*' OR 'cadaveric donor*' OR 'cadaveric kidney donor*' OR 'cadaveric renal donor*' OR 'cadaveric organ donor*' OR 'donor'/exp OR 'kidney donor'/exp) AND ('randomized controlled trial':it OR 'controlled clinical trial':it OR 'randomized':ti,ab,kw OR 'placebo':ti,ab,kw OR 'drug therapy' OR 'randomly':ti,ab,kw OR 'trial':ti,ab,kw OR 'groups':ti,ab,kw)

COCHRANE: ("Kidney Transplantation" OR "Kidney graft" OR "Renal graft" OR "Renal Transplantation" OR "Kidney Transplant Surgery" OR "Renal Transplant" OR "Kidney Transplant" OR "Kidney Donor*") AND ("termed targeted temperature management" OR "targeted temperature management" OR "Therapeutic Hypothermia" OR Hypothermia OR "Cooling therapy")

Adverse Events per Study:

Table S1 - Donor's Adverse Events per Study

Study	Adverse Events per Study Group	
	**Hypothermia**	**Normothermia**
**HYPOREME, 2024**	2 Cardiac Arrythmias ; 2 Cardiac Arrests	3 Cardiac Arrythmias ; 2 Cardiac Arrests
**Malinoski, 2023**	6 Cardiac Arrythmias ; 2 Hypotensions ;	1 Cardiac Arrest
**NMA, 2015-2019**	1 Cardiac Arrythmia ; 1 Systemic Hypertension	2 Cardiac Arrests
**Patel, 2024**	Not Reported	Not Reported

Results Supplement

Additional Information of Included Studies, Donors and Recipients:

Table S2 – Additional Information not reported in Table 1

Included Studies' Characteristics				
	**Malinoski 2019**	**NMA 2015-2019**†	**Patel 2024**	**HYPOREME 2024**
**Hypothermia Definition**	34–35°C	34–35°C	34–35°C	34–35°C
**Normothermia Definition**	36.5–37.5°C	36.5–37.5°C	36.5–37.5°C	36.5–37.5°C
**Follow-up Time**	1 year	1 year	1 year	1 year
**Donor's Baseline Characteristics**				

	Malinoski 2019	NMA 2015-2019†	Patel 2024	HYPOREME 2024
**Prior Treatment with Hypothermia**	93(19%) / 66(13%)	18(12%) / 18(11.8%)	53(22%) / 43(18%)	NA
**Creatinine at enrollment (mg/dl)**	1.37±0.84 / 1.34±0.86	1.1±0.6 / 1.1±0.6	0.99±0.43 / 1.07±0.6	0.93±0.45 / 0.92±0.49‡
**GFR at enrollment (mL/min/1.73 m²)**	77±34 / 80±36	89.2±50.8 / 89.0±43.1	101.26±33.92 / 96.45±31.51	76.1±22.3 / 77.6±22.6
**GFR before Transplant/Procurement (mL/min/1.73 m²)**	94±40 / 84±39	103.4±58.1 / 88.2±43.9	113.78±34.03 / 101.19±32.51	76.5±23.8 / 74.1±24.2

Data is either reported as mean ± SD, No. (%), or median (IQR). GFR = Glomerular filtration rate, †NMA = Niemman 2015, Malinoski 2019, Axelrod 2019, which were three different publications of a same research ‡originally reported in µmol/L, converted to mg/dl by dividing the original value by 88.42 and rounding to two decimal cases.

Primary Outcomes Additional Subgroup Analyses:

Figure S1 – Delayed graft function (DGF) with Donor's Therapeutic Hypothermia vs Normothermia including only USA studies

Study or Subgroup	Experimental Events	Experimental Total	Control Events	Control Total	Weight	Risk ratio M-H, Random, 95% CI
Malinoski, 2023	103	479	99	510	33.9%	1.11 [0.87 , 1.42]
NMA, 2015-2019	79	280	112	286	34.7%	0.72 [0.57 , 0.91]
Patel, 2024	79	460	87	474	31.4%	0.94 [0.71 , 1.23]
**Total (95% CI)**		**1219**		**1270**	**100.0%**	**0.90 [0.70 , 1.17]**
Total events:	261		298			

Heterogeneity: Tau² = 0.04; Chi² = 6.25, df = 2 (P = 0.04); I² = 68%
Test for overall effect: Z = 0.76 (P = 0.45)
Test for subgroup differences: Not applicable

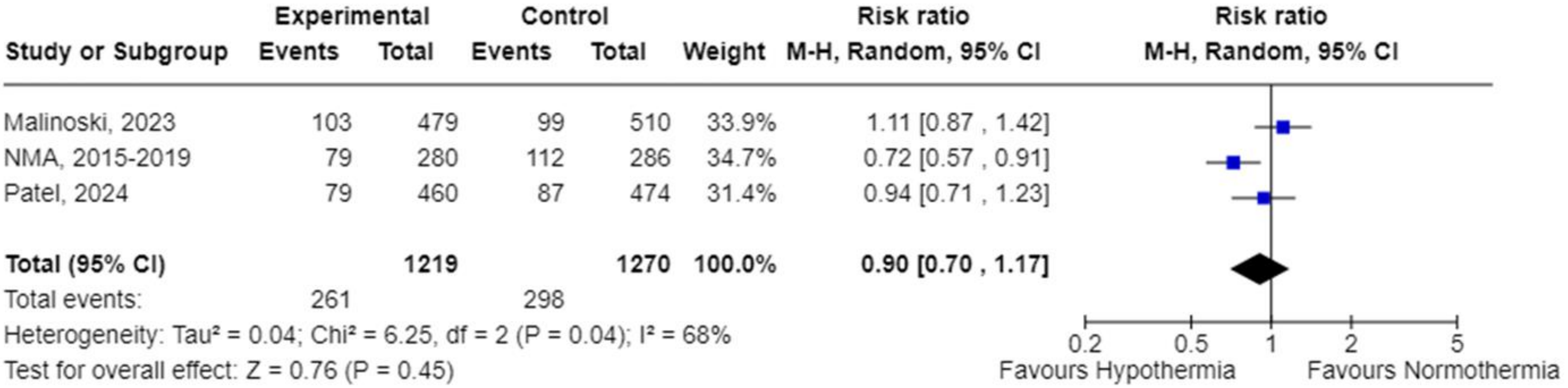

Figure S2 – Graft Failure with Donor's Therapeutic Hypothermia vs Normothermia including only USA studies

Study or Subgroup	Experimental Events	Experimental Total	Control Events	Control Total	Weight	Risk ratio M-H, Random, 95% CI
Malinoski, 2023	13	475	17	509	44.6%	0.82 [0.40 , 1.67]
NMA, 2015-2019	14	281	24	284	55.4%	0.59 [0.31 , 1.12]
**Total (95% CI)**		**756**		**793**	**100.0%**	**0.68 [0.42 , 1.10]**
Total events:	27		41			

Heterogeneity: Tau² = 0.00; Chi² = 0.46, df = 1 (P = 0.50); I² = 0%

Test for overall effect: Z = 1.57 (P = 0.12)

Test for subgroup differences: Not applicable

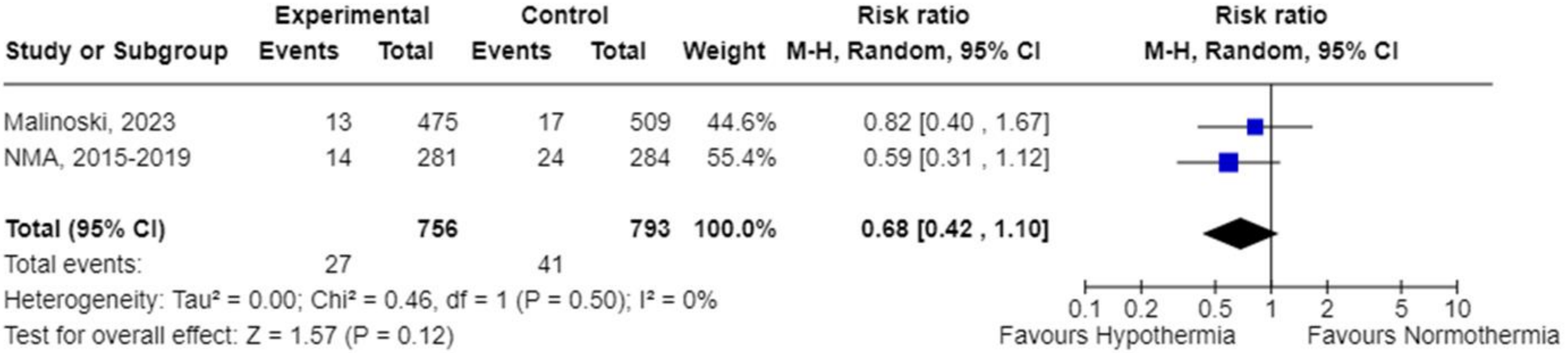

Secondary Outcomes Analyses:

Figure S3 – Recipient's Mortality with Donor's Therapeutic Hypothermia vs Normothermia

	Hypothermia		Normothermia			Risk ratio
**Study or Subgroup**	**Events**	**Total**	**Events**	**Total**	**Weight**	**M-H, Random, 95% CI**
HYPOREME, 2024	15	251	20	275	48.8%	0.82 [0.43 , 1.57]
Malinoski, 2023	18	479	19	510	51.2%	1.01 [0.54 , 1.90]
**Total (95% CI)**		**730**		**785**	**100.0%**	**0.91 [0.58 , 1.43]**
Total events:	33		39			

Heterogeneity: $Tau^2 = 0.00$; $Chi^2 = 0.20$, df = 1 (P = 0.66); $I^2 = 0\%$

Test for overall effect: Z = 0.40 (P = 0.69)

Test for subgroup differences: Not applicable

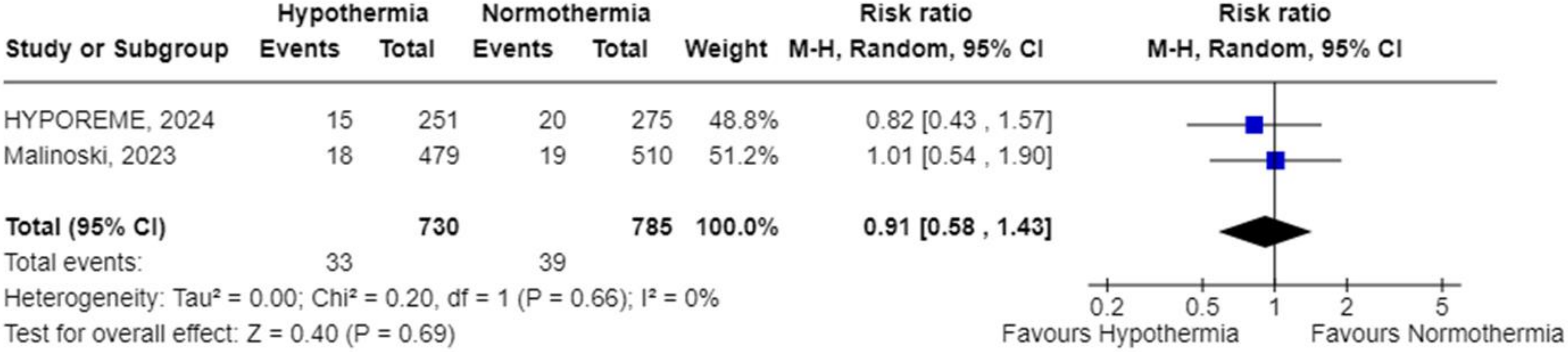

Figure S4 – Donor’s Adverse Events with Donor’s Therapeutic Hypothermia vs Normothermia

	Hypothermia		Normothermia			Risk ratio
Study or Subgroup	Events	Total	Events	Total	Weight	M-H, Random, 95% CI
HYPOREME, 2024	4	183	5	182	51.4%	0.80 [0.22 , 2.92]
Malinoski, 2023	8	838	1	510	22.9%	4.87 [0.61 , 38.81]
NMA, 2015-2019	2	150	2	152	25.7%	1.01 [0.14 , 7.10]
**Total (95% CI)**		**1171**		**844**	**100.0%**	**1.28 [0.45 , 3.62]**
Total events:	14		8			

Heterogeneity: $Tau^2 = 0.11$; $Chi^2 = 2.27$, df = 2 (P = 0.32); $I^2 = 12\%$
Test for overall effect: Z = 0.47 (P = 0.64)
Test for subgroup differences: Not applicable

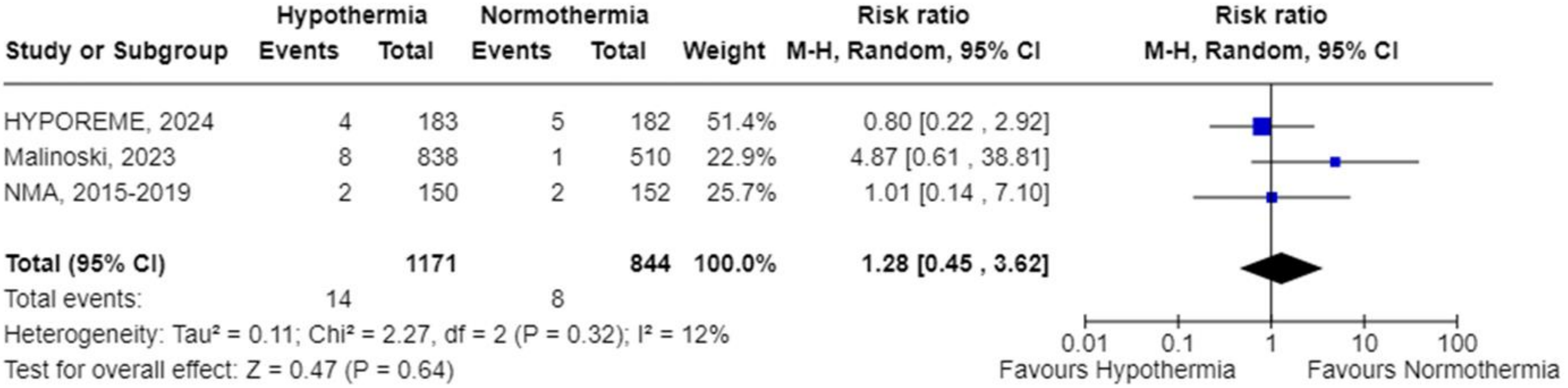

Heterogeneity Analysis:

Figure S5 – Contribution to Overall Heterogeneity – DGF Baujat Plot

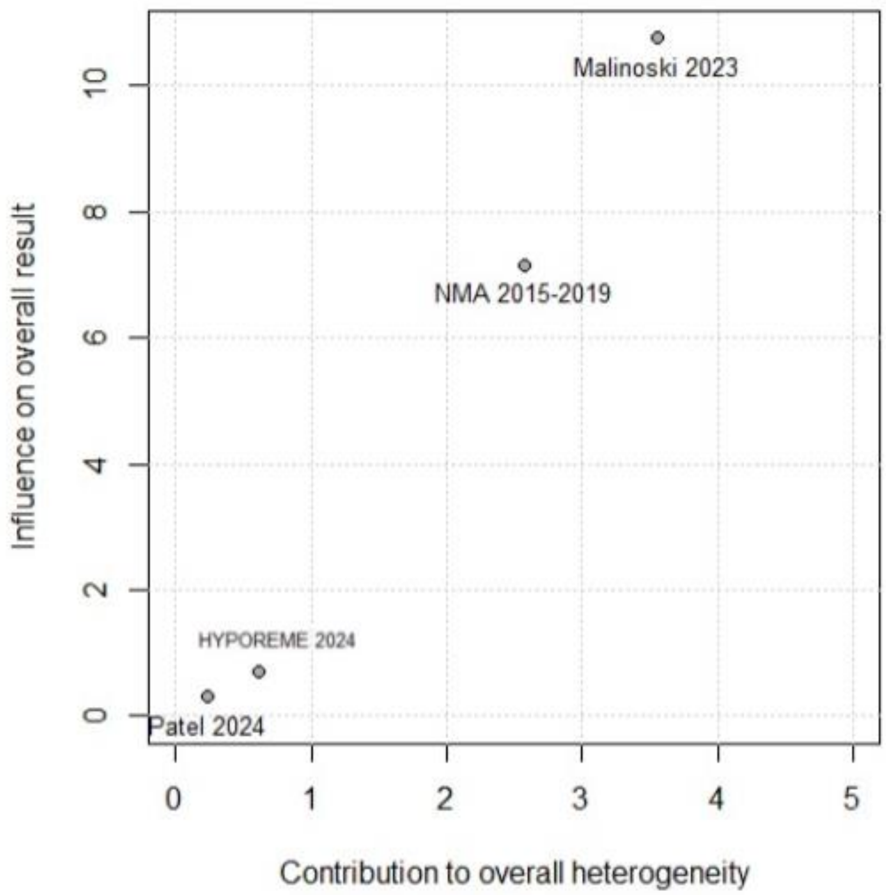

Sensitivity Analysis – Leave-one-out method:

Figure S6 – Sensitivity Analysis (Leave-one-out) – DGF

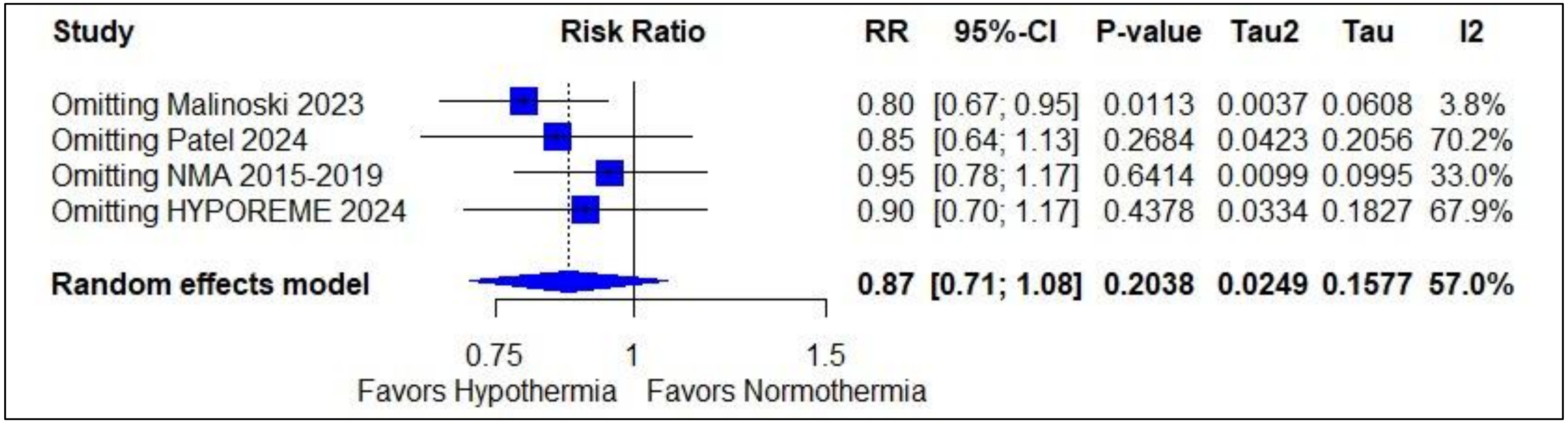

Figure S7 – Sensitivity Analysis (Leave-one-out) – Graft Failure

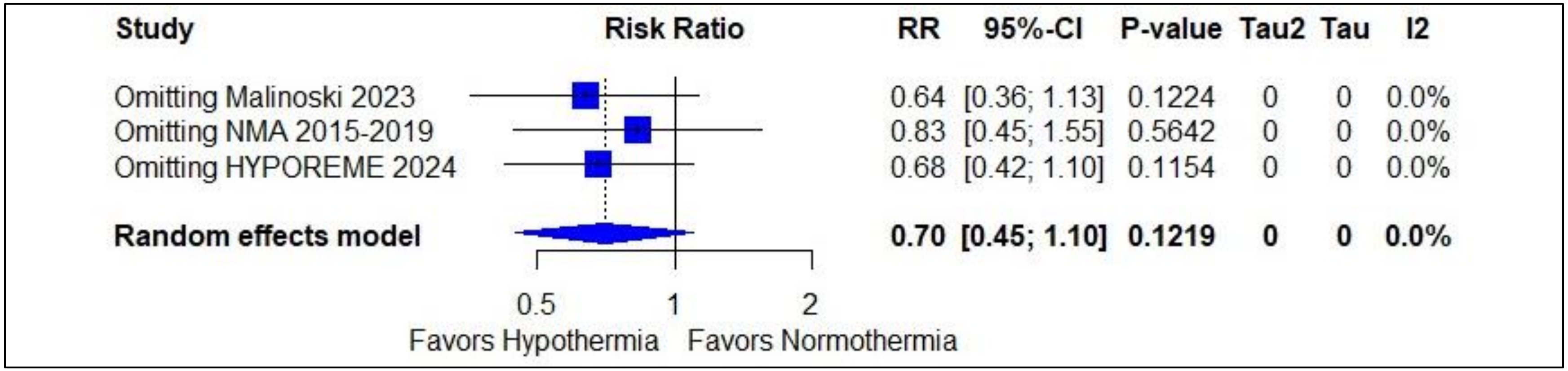

Risk of Bias Assessment:

Table S3 – RoB-2 Bias Assessment of Included Studies

Study	Bias from randomization process	Bias due to deviations from intended interventions	Bias due to missing outcome data	Bias in measurement of the outcomes	Bias in selection of the reported result	Overall Bias
**Malinoski 2023**	**Some concerns**	**High**	**Low**	**Low**	**Low**	**High**
**NMA 2015-2019†**	**Some concerns**	**Low**	**Low**	**Low**	**Low**	**Some concerns**
**Patel 2024**	**Low**	**Low**	**Low**	**Low**	**Some concerns**	**Some concerns**
**HYPOREME 2024**	**Low**	**Some concerns**	**Low**	**Low**	**Low**	**Some concerns**

†NMA = Niemman 2015, Malinoski 2019 and Axelrod 2019, which were three different publications of a single randomized controlled trial
